# Cation-Dependent Interfacial Properties Determine the Activity of Pt(111) Electrodes in Alkaline Media

**DOI:** 10.1021/acscatal.5c05622

**Published:** 2025-11-12

**Authors:** Haiting Yu, Song Xue, Elena L. Gubanova, Jian Zhou, Rodrigo Bautista, Adrian V. Himmelreich, Aliaksandr S. Bandarenka

**Affiliations:** † Physics of Energy Conversion and Storage, Department of Physics, 9184 Technical University of Munich, James-Franck-Str. 1, 85748 Garching bei München, Germany; ‡ Research Center on Advanced Chemical Engineering and Energy Materials, 71136 China University of Petroleum (East China), Qingdao 266580, P. R. China; § Catalysis Research Center TUM, Ernst-Otto-Fischer-Str. 1, 85748 Garching bei München, Germany

**Keywords:** electrode−electrolyte interface, Pt single crystal, interfacial properties, alkali metal cations, reaction descriptor, interfacial entropy, electrocatalytic activity

## Abstract

Energy conversion and storage technologies require optimal electrode–electrolyte interfaces to drive electrocatalytic reactions. However, the impact of interfacial phenomena on the catalytic activity remains debated. This study investigates the role of alkali metal cations in interfacial properties and correlates them with electrocatalytic activities toward several energy-related reactions in alkaline media using model Pt(111) single crystal electrodes. Through electrochemical impedance spectroscopy and laser-induced current transient techniques, interfacial parameters, such as the double layer capacitance, the potential of the capacitance minimum, and the potential of maximum entropy (pme), are determined. The latter exhibit a linear dependence on cation hydration energies. Notably, two distinct pmes are observed at the Pt(111)-alkaline electrolyte interfaces, attributed to water dipole reorientation. Correlating pme with reaction activities reveals that interfacial entropy is a robust and general descriptor of electrocatalytic reaction kinetics. Particularly, electrocatalytic activity improves as the pme aligns more closely with the thermodynamic equilibrium potential of the respective reaction, providing a solid framework for optimizing interfacial microenvironments to enhance electrocatalytic performance.

## Graphical abstract

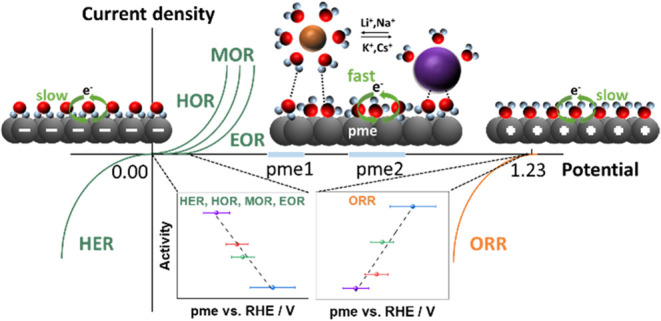

## Introduction

The transition to sustainable development is imperative to address global challenges of climate change and the energy crisis caused by excessive fossil fuel consumption. Renewable energy technologies, including fuel cells, electrolyzers, and batteries, offer cleaner alternatives, supporting the reduction of greenhouse gas emissions and sustainable energy provision. Heterogeneous electrocatalysis underpins these technologies, driving reactions such as hydrogen evolution and oxidation (HER/HOR),^1,2^ oxygen evolution/and reduction (OER/ORR),^3,4^ carbon dioxide reduction (CO_2_RR)^5,6^ or nitrate reduction (NO_3_RR).^7^ However, the efficiency of those reactions is usually limited by sluggish kinetics and high reaction barriers due to the complex interactions between adsorbates, electrolyte components, and the catalyst surface, as well as the multiple electron transfer steps involved.

The electrified electrode–electrolyte interface plays a crucial role in understanding and optimizing the catalytic activity.^8–73^ To unravel its complexity, a variety of in situ monitoring techniques, such as X-ray^12–15^ and vibrational spectroscopies,^16–21^ have been developed to probe interfacial properties, particularly the structure of interfacial water. Recent studies have highlighted the importance of electric double layer (EDL) characteristics in controlling reaction kinetics and mechanisms.^22,23^ For instance, hydrogen binding energy (HBE) primarily dictates HER activity in acidic media,^24^ while hydroxyl adsorption strength^25^ and interfacial electric field^26^ are essential factors in alkaline environments. Moreover, ion-intermediate coordination has been recognized as a catalytic activity descriptor for CO_2_RR, and interfacial hydrogen bonding networks dominate proton-coupled electron transfer reactions.^27–29^ Other factors, such as electrostatic interactions, oxophylicity of reactive sites, and water dynamics, also significantly influence reaction kinetics.^30–32^ Importantly, electrolyte composition, especially the nature of alkali metal cations, profoundly affects interfacial properties. These cations modulate the EDL structure through their intrinsic nature in terms of size, electronegativity, hydration energy, solvation degree, and Lewis acidity, thereby tailoring the activity, selectivity, and Faradaic efficiency of electrocatalytic reactions.^33–36^ Despite remarkable progress, molecular-level insights into the cation-dependent interfacial microenvironment under operando conditions remain challenging, particularly concerning interfacial entropy and its relationship with reaction kinetics.

To address these gaps, we present a quantitative evaluation of various cation-dependent interfacial parameters on a Pt(111) single crystal electrode in alkaline media. This is accomplished by combining electrochemical impedance spectroscopy (EIS) and a unique methodology, the in situ laser-induced current transient (LICT) technique. Furthermore, we establish a direct correlation between the interfacial microenvironment properties and the activity of several energy-related electrocatalytic reactions, proposing a compelling and general catalytic activity descriptor that has been successfully validated through experiments and literature data.

## Experimental Section

### Chemicals

All chemicals used in this study were of the highest purity available. Diluted perchloric acid solutions (70% HClO_4_, Suprapur, Merck, Germany) were prepared using ultrapure water (18.2 MΩ·cm, Merck Millipore, Germany). Alkaline electrolytes were prepared from LiOH (99.995%, Thermo Scientific, Germany), NaOH (99.99%, Sigma-Aldrich, Germany), KOH (99.99%, Sigma-Aldrich, Germany), and CsOH (99.95%, Sigma-Aldrich, Germany) and diluted to 0.1 M concentration.

### Electrochemistry

Electrochemical measurements were performed in a three-electrode configuration with a Pt(111) single-crystal disk electrode (10 mm, MaTecK, Germany) as the working electrode, a Pt wire as the counter electrode, and either a mercury-mercurous sulfate (MMS) or a mercury-mercurous oxide (MMO) electrode as the reference. A VSP-300 potentiostat (BioLogic, France) controlled the experiments, with all potentials referenced to the reversible hydrogen electrode (RHE). The Pt(111) electrode was cleaned via electrochemical cycling in 0.1 M HClO_4_, followed by flame annealing and cooling in a CO/Ar gas mixture. Surface quality was verified through cyclic voltammetry (CV) in Ar-saturated 0.1 M HClO_4_.

### Electrochemical Impedance Spectroscopy (EIS)

Staircase potentiostatic EIS measurements were conducted in the AC frequency range from 300 kHz to 1 Hz with a perturbation amplitude of 10 mV and a potential step width of 25 mV. A shunt capacitor (∼10 μF) was connected between the reference and counter electrodes to address high-frequency artifacts. Impedance data were analyzed using custom software,^37^ with the data validated through a Kramers–Kronig check.

### Laser-Induced Current Transient (LICT) Experiments

LICT experiments were performed using a Quanta-Ray INDI Series Pulsed Nd:YAG laser (532 nm, 5–8 ns pulse duration, Spectra Physics, USA). The beam intensity was attenuated with a motorized beam splitter to prevent damage to the electrode. The laser pulses were guided through a quartz window of a custom-made three-electrode cell to illuminate the Pt(111) electrode. Chronoamperometry was used to record current transients with a potential step width of 20 mV.

## Results and Discussion

The Pt(111) electrode was initially characterized in 0.1 M HClO_4_ solution to verify the high quality and cleanliness of its surface. The acquired cyclic voltammograms (CVs) (Figure 1a) demonstrate excellent reproducibility, providing a robust basis for the reliable interpretation of the adsorption dynamics under investigation.^38^ Subsequently, CVs were recorded in Ar-saturated 0.1 M AMOH electrolytes containing different alkali metal cations (AM = Li^+^, Na^+^, K^+^, Cs^+^), as presented in Figure 1b. The CVs exhibit three distinct potential regions: region I (0.05–0.4 V) is associated with hydrogen underpotential desorption (H^+^ + e^–^ ↔ H_upd_); region II (0.4–0.65 V) corresponds to double-layer charging and discharging; and region III (0.65–0.85 V) is attributed to reversible OH adsorption and desorption (OH^–^ ↔ OH_ad_ + e^–^). Notably, while the H_upd_ and double-layer charging profiles remain largely unaffected by the nature of the electrolyte cation, a pronounced cation effect is observed in the OH adsorption region. Both the onset potential and the main peak of OH adsorption shift negatively in the series Cs^+^ > K^+^ ≈ Na^+^ > Li^+^, providing direct electrochemical evidence that alkali metal cations influence the formation of the OH_ad_ layer on Pt(111). This trend, most pronounced in Li^+^-containing electrolytes, is consistent with recent studies attributing such effects to noncovalent interactions between hydrated alkali metal cations accumulated at the outer Helmholtz plane (OHP) rather than specifically adsorbing onto the electrode surface and adsorbed OH-species at the interface.^39,40^ The inverse correlation between the cation hydration energy (Li^+^ > Na^+^ > K^+^ > Cs^+^) and the shift in OH_ad_ potential is a critical clue. It suggests that the more strongly hydrated cations, which possess a well-structured hydration shell, more effectively stabilize or less destabilize the adsorbed OH species through their oriented water networks. This long-range, water-mediated interaction lowers the energy of the OH_ad_ state, facilitating its formation at more negative potentials without requiring the cation to lose its solvation shell and specifically adsorb. Consequently, this leads to a relative increase in OH_ad_ coverage compared to electrolytes containing less strongly hydrated cations.^41–43^

**Figure 1:**
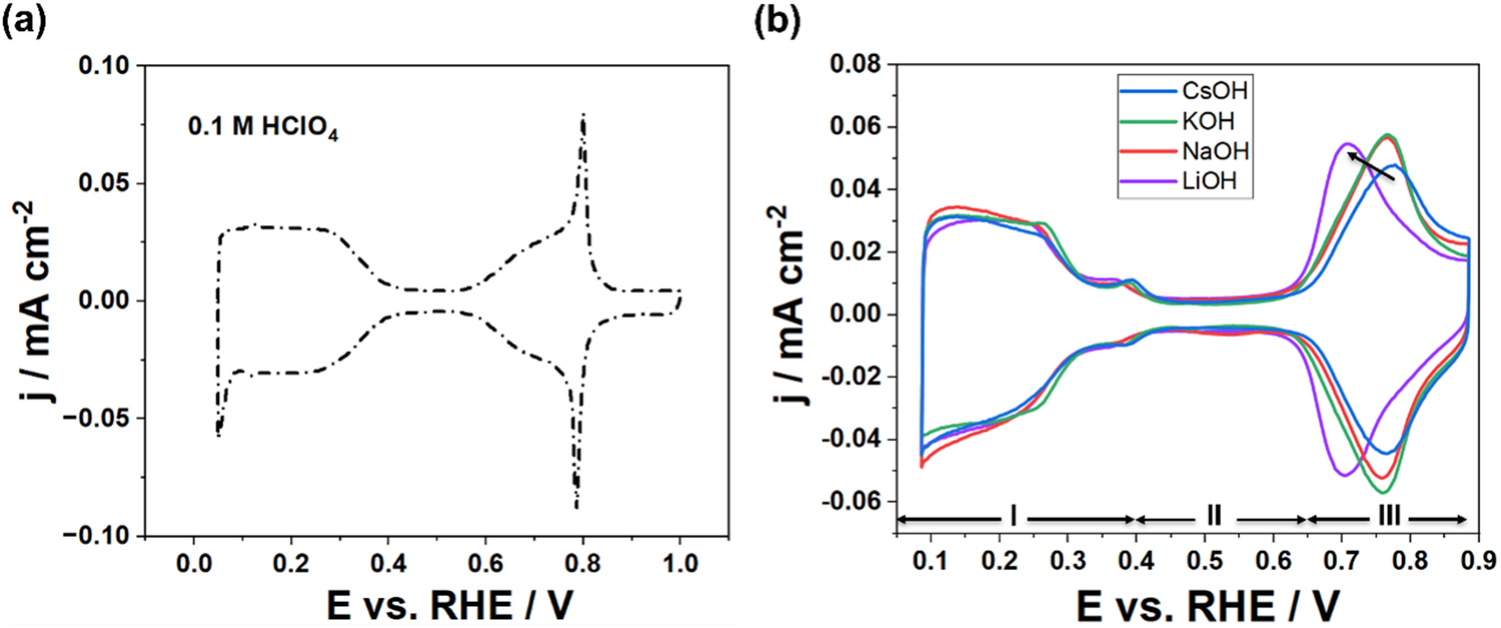
CVs of Pt(111) in Ar-saturated (a) 0.1 M HClO_4_ solution and (b) 0.1 M alkaline electrolytes containing different cations, recorded at a scan rate of 50 mV/s. CVs in alkaline electrolytes include three potential regions: H_upd_ in region I, the double layer charging and discharging in region II, and the reversible OH-adsorption in region III. The black arrow indicates the effect of cations on OH-adsorption.

This interpretation, however, must be reconciled with important studies proposing that cations can influence interfacial processes through mechanisms such as site-blocking or specific adsorption. We suggest that these views are not mutually exclusive but represent different manifestations of cation effects, with the dominant behavior being controlled by three key interfacial conditions:

1. Potential regime. The observed noncovalent effects dominate at the moderate anodic potentials relevant to the formation of the OH_ad_ layer on a pristine Pt(111) surface. In this potential window, the electric field is sufficient to attract cations to the OHP but insufficient to strip the hydration shell of small cations like Li^+^. In contrast, studies reporting site-blocking often focus on very different potential regimes. For instance, research on the hydrogen evolution or CO_2_ electroreduction^44,45^ operates at significant cathodic potentials, where the immense negative surface charge generates a powerful electric field and overcome the hydration energy of cations and promote partial desolvation, potentially enabling closer approach or partial specific adsorption.
2. Electrode surface structure (defects) and composition. Terrace sites on well-ordered Pt(111) surface have a homogeneous and relatively weak local electric field, favoring the accumulation of fully hydrated cations at the OHP. However, stepped surfaces and defects with low coordination number generate stronger local electric fields that can lower the energy barrier for cation desolvation, promoting a transition from nonspecific accumulation to specific adsorption and site-blocking behavior.^46^ Furthermore, the base metal itself (e.g., Au vs Pt) has a different work function and surface chemistry, which also influences cation behavior.^47^
3. Electrolyte concentration and pH. Higher concentration and pH cause higher near-surface cation concentration, might inducing the blockage effect.^48^

The voltammetric signature in our work is a clear indicator of noncovalent, water-mediated interactions originating from hydrated cations in the OHP under our experimental conditions. The apparent contradiction with studies about site-blocking or specific adsorption is not a fundamental conflict but a reflection of the dynamic nature of the electrochemical interface. Rather than separate phenomena, these two effects represent different points on a continuum of cation-surface interactions. The operative mechanism is dictated by the balance between a cation hydration energy and the energy supplied by the interfacial environment. By applying a more extreme potential, introducing defect sites, or altering the electrolyte, the interfacial microenvironment can be shifted along this continuum, from long-range hydration-shell effects to short-range site-blocking behavior.

EIS analyses were executed to probe the effects of alkali metal cations on the EDL characteristics and electrocatalytic processes involving mass and charge transfer.^74,75^ All measurements were conducted in regions where continuous Faradaic reactions were absent. The impedance spectra were analyzed using an equivalent electric circuit (EEC) model (Figure 2a), which includes a constant phase element (CPE) to accurately describe the double layer response.^49^ The CPE impedance is given by *Z*_dl_ = 1/*C*_dl_′(*j*ω)^−*n*^, where the CPE exponent *n* indicates the deviation from ideal double layer behavior, and *C*_dl_′ is interpreted as the *C*_dl_ when the exponent *n* is close to unity. Further impedance analysis details are presented in the Supporting Information (SI).

**Figure 2:**
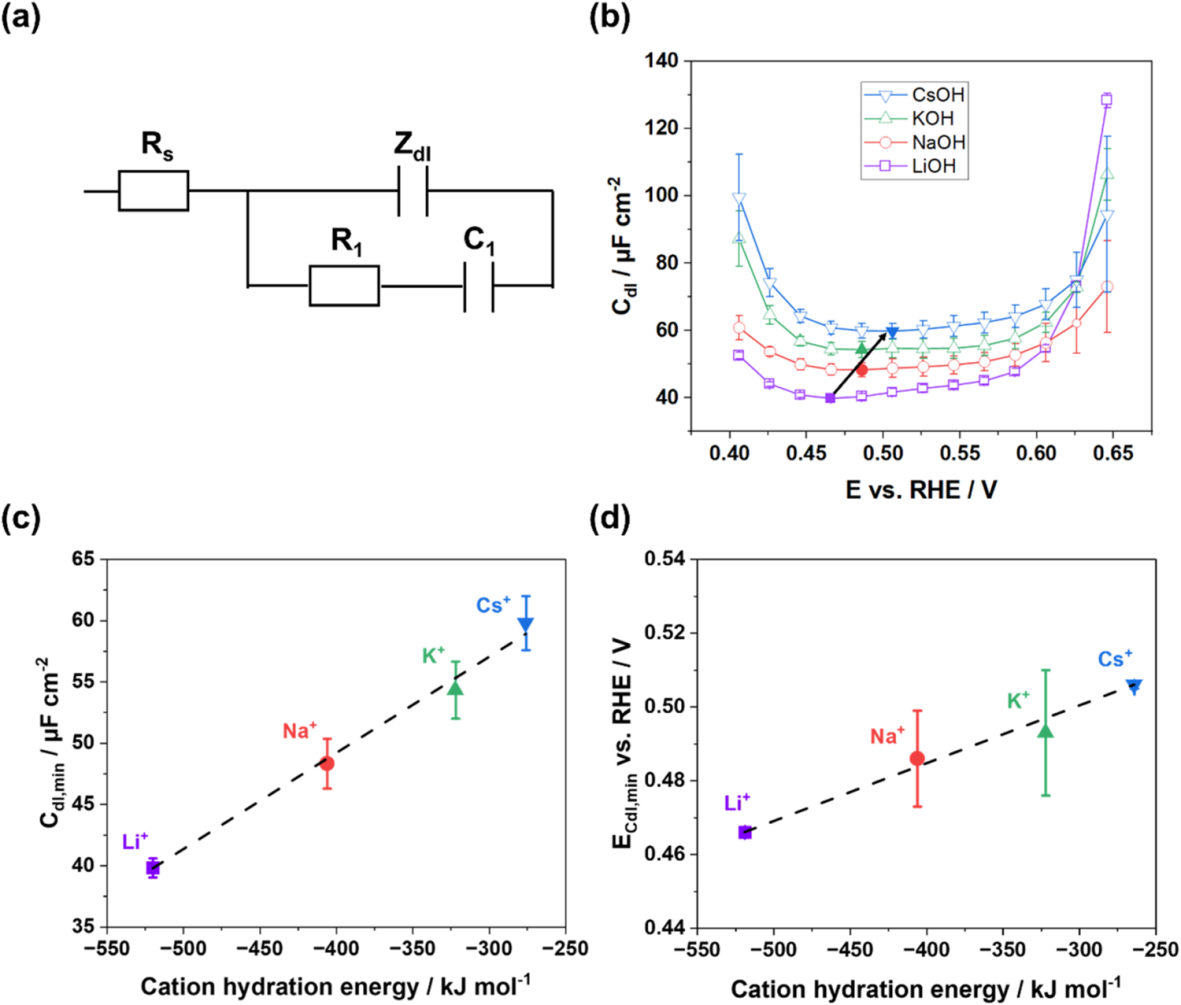
(a) EEC model employed to fit the impedance spectra, including uncompensated resistance *R*_s_, double layer impedance *Z*_dl_, adsorption resistance *R*_1_, and capacitance *C*_1_. (b) The dependence of *C*_dl_ on applied potential within the double layer region for different cation solutions; filled points mark the minimum for each solution. (c) *C*_dl,min_ extracted from (b) and plotted as a function of the hydration energy of the alkali metal cations. (d) The relationship between *E*_Cdl,min_ and the cation hydration energy. Linear fits are shown with dashed black lines in (c, d).

The *C*_dl_(*E*) dependence for Pt(111) in four different 0.1 M AMOH electrolytes is illustrated in Figure 2b. Notably, *C*_dl_ increases consistently in the order LiOH < NaOH < KOH < CsOH across the entire double layer potential region. At more positive potentials (*E* > 0.6 V), a sharp increase in *C*_dl_ is observed for LiOH that can be attributed to the rapid OH-adsorption. Figure 2c reveals a strong linear relationship between the minimum capacitance (*C*_dl,min_) and the hydration energy of the alkali metal cations. This trend, where *C*_dl,min_ increases with decrease of cation hydration, is consistent with previous studies across various electrode materials (compared among Pt(111), Au(111) and Cu(111) electrodes) and electrode surface orientations (compared among Pt(111), Pt(775) and Pt(12 10 5) electrodes).^50^ The observed variation in *C*_dl_, from ∼40 μF/cm^2^ in LiOH up to ∼60 μF/cm^2^ in CsOH, is attributed to the nature of the alkali metal cations. The smaller intrinsic ionic radius of Li^+^ gives rise to its high charge density and strong hydration energy, whereas the larger Cs^+^ ion, with lower charge density, exhibits much weaker hydration and is prone to partial desolvation. Consequently, structure-making cations such as Li^+^ and Na^+^, with strong water–cation interactions, tend to preserve their solvation shells and stabilize a well-organized hydrogen-bonding network within the EDL. In contrast, structure-breaking cations such as K^+^ and Cs^+^, with weaker hydration, more readily disrupt the interfacial H-bonding network and can dynamically approach the electrode surface.^51–53^

These contrasting behaviors give rise to two main mechanisms underlying the observed cation-dependent *C*_dl_ trends:

1. Cation-surface distance: Larger, partially desolvated cations (K^+^ and Cs^+^) accumulate closer to the electrode surface by displacing interfacial water molecules. The shortened cation–surface separation decreases the effective double-layer thickness, thereby increasing C_dl_, as supported by molecular dynamics simulations.^54^
2. Relative permittivity: the strong charge density of Li^+^ enhances local interfacial polarity and stabilizes higher OH_ad_ coverage, increasing the effective number concentration of strongly hydrated ions near the electrode. This enforces a rigid hydrogen-bonding network that is less responsive to electrode polarization, lowering the local relative permittivity (ε_r_). The reduced ε_r_ collectively suppresses *C*_dl_, consistent with DFT and other theoretical studies.^55^

Figure 2d presents a linear relationship between cation hydration energy and the potential of the capacitance minimum (*E*_Cdl,min_), which shifts positively from 0.466 to 0.506 V as the hydration energy decreases (Li^+^ < Na^+^ < K^+^ < Cs^+^). This trend signifies that less-hydrated cations like Cs^+^ stabilize a water structure that resembles the interface at a more positive applied potential. This is consistent with the concept that the larger and less-hydrated cations accumulate more readily near the Pt(111) surface and can more effectively disrupt the H-bonding network of water molecules, thereby altering the interfacial charging distribution and structure. This cation-induced reconfiguration directly modulates the interfacial electric field, which governs the alignment of ions, water dipoles, and other adsorbates, controlling the overall interfacial environment.^56^ Consequently, these alterations directly impact crucial electrochemical processes, including ion adsorption kinetics, electron transfer rates, proton dynamics, and ultimately, the catalytic efficiency of key reactions.

Collectively, these impedance results shed light on the importance of understanding ion-specific behavior at the interface for the rational design of more effective catalyst-electrolyte interfaces in energy conversion processes.

To further elucidate how interfacial structure and solvation environment correlate with the activity of electrocatalytic reactions, we conducted in situ LICT experiments. This technique utilizes short-pulsed laser irradiation to rapidly elevate the interfacial temperature, momentarily perturbing the structured water layer. During the relaxation period, sharp current transients are monitored, reflecting variations in the double layer restructuring. The sign and magnitude of these transients provide information about the solvent molecules’ orientation and degree of disorder at the interface. Notably, the potential at which the current transients vanish or change sign is identified as the potential of maximum entropy (pme), representing a state of maximum interfacial disorder, where no net dipolar contribution is present. This parameter is crucial for understanding the looseness of the water layer and the interfacial reorganization energy involved in charge and mass transfer during electrocatalytic reactions.

The integrated charge densities from the current transients for four alkaline electrolytes as a function of applied potential are plotted in Figure 3a–d. The interpretation of LICT signals on metal electrodes requires careful discrimination between Faradaic (adsorption or desorption) and non-Faradaic (double-layer reordering) contributions. Based on the following evidence, we attribute the observed transients predominantly to the reorientation of water molecules:

1. Exclusion of H_upd_ kinetics. The contribution of H_upd_ to the fast transient signal is negligible. This assignment is confirmed by our analysis of the transient relaxation times, which are consistently on the order of ∼0.16 μs across applied potentials and electrolytes (as shown in Figure S3). This time scale is orders of magnitude faster than the characteristic millisecond-to-second kinetics reported for H_upd_ kinetics,^57^ ruling it out as the governing process for our signals. This kinetic argument for excluding H_upd_ is well-supported in the literature.^58,59^

2. Exclusion of OH adsorption kinetics. We can also rule out a dominant contribution from hydroxide adsorption (Pt + OH^–^ → Pt–OH + e^–^). This is generally an exothermic process.^60^ The laser-induced temperature jump would therefore shift the equilibrium to favor desorption, a reaction that releases electrons into the external circuit, generating positive current transients. The consistent absence of such positive transients in the potential region associated with OH adsorption allows us to exclude it. Instead, the negative transients we observe are characteristic of water reorientation.

**Figure 3:**
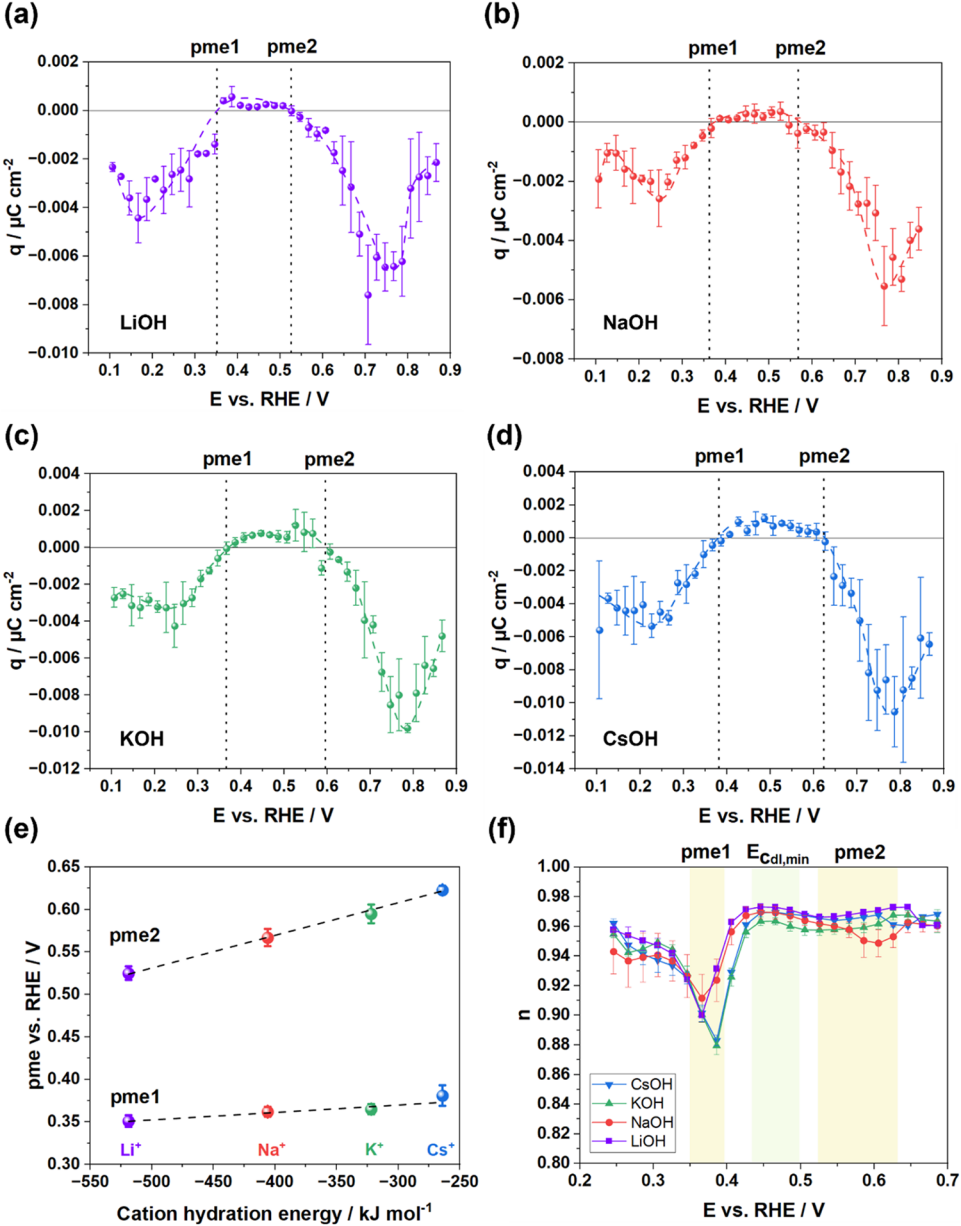
(a–d) Exemplary charge densities obtained from the integration of the current transients versus potential in 0.1 M AMOH electrolytes. The corresponding dashed line is inserted as a guide to the eye, and the dashed black line indicates the obtained pme values. (e) Two pme values derived from (a–d) for each electrolyte, plotted as a function of the cation hydration energy. (f) The *n*-dependences on the potential.

Consequently, the responses across the entire potential window are dominated by the reorientation of water molecules.

Analysis of charge densities reveals negative values in the H_upd_ region, consistent with a net H-down water orientation. As the potential increases and this ordered structure destabilizes, the interfacial entropy rises, reaching a maximum at the transition between the H_upd_ region and the double layer region. This pme signifies the potential where the H-down configuration has become sufficiently disordered. Within the double layer region, the charge density becomes positive, signifying a more structured water layer. A second pme is observed at the onset of OH-adsorption, reflecting further dipole reorientation. With increasing potential, the extent of water orientation initially rises but decreases beyond *E* > 0.8 V. The extensive formation of OH_ad_ and O_ad_ imposes a new, more ordered configuration on the interface,^76^ might leading to a decrease in the overall water orientation dynamics observed in the LICT signal. Figure 3e discloses a linear dependence of both pme values on the hydration energy of alkali metal cations, a trend similar to that of the *E*_Cdl,min_. Combining these results with the *n*-dependence data from EIS experiments (Figure 3f), we observe that n approaches 1 near the *E*_Cdl,min_.^61^ A pronounced minimum in *n* is evident within the first pme region, with its position shifting to more positive potentials for larger cations, in accordance with the shift in the pme value. Additionally, a local minimum in n appears within the second pme region. These sharp drops and local minima are attributed to interfacial heterogeneities arising from the structuring and restructuring of water molecules at the interface with hydrogen bond breaking.^62^

Overall, the EIS and LICT results provide complementary, operando evidence that alkali metal cations tune the interfacial electric field and hydrogen-bonding network. The cation-dependent shifts in *E*_Cdl,min_ and the pme directly reflect how cations alter the energy landscape for water reorientation and dipole alignment, thereby regulating the degree of interfacial disorder and ultimately governing electrocatalytic efficiency.

Previous studies from our group have demonstrated that the kinetics of electrocatalytic reactions are closely linked to cation-dependent pme values. This relationship holds for both single-crystal and polycrystalline electrodes^64,65^ and extends to more complex electrode systems.^66,67^ In the vicinity of the pme, the interfacial environment becomes highly disordered, facilitating the rearrangement and reorientation of interfacial components during mass and charge transfer, which accelerates electrocatalytic reactions. Consequently, it can be inferred that reaction activity improves as the pme moves closer to the respective reaction’s thermodynamic equilibrium potential (TEP). To validate this hypothesis, we compared pme values with activity measurements for a series of energy-related electrocatalytic reactions, focusing on those catalyzed by Pt(111), which are relevant to a variety of applications, as summarized in Figure 4. For HER and HOR, both having an identical TEP of 0 V, activity increases linearly with decreasing pme values. Notably, LiOH demonstrates pme values closest to 0 V, correlating with higher activity. Conversely, for ORR, with a TEP of 1.23 V, activity rises linearly with increasing pme values, with CsOH displaying pme values nearest to the ORR TEP. These reactions are paramount for water electrolysis and hydrogen fuel cell technologies. Moreover, the methanol oxidation reaction (MOR) and ethanol oxidation reaction (EOR) were also investigated, as they are fundamental to the direct methanol and ethanol fuel cells (DMFCs and DEFCs), electrosynthesis, and other energy-related technologies.^68,69^ The TEPs of both reactions are similar to that of HOR, lying at 0.016 and 0.084 V, respectively.^70^ Remarkably, the activities of these two reactions follow a trend similar to that of HOR, with reaction activity increasing linearly as pme values decrease.

**Figure 4:**
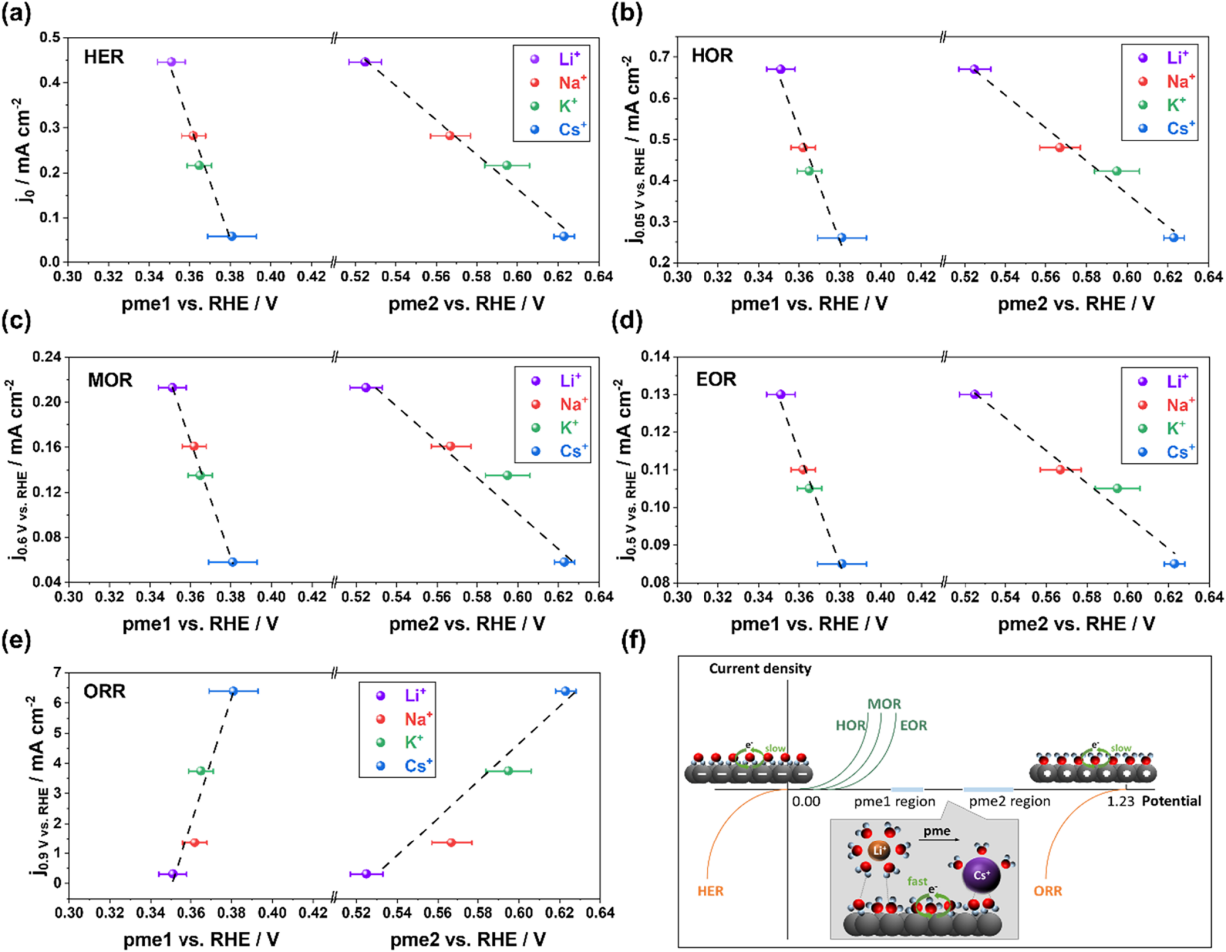
Multiple energy-related catalytic reactions are involved: the exchange current densities of HER vs two pme values shown in (a); the current densities of HOR at 0.05 V vs two pme values shown in (b); the current densities of MOR at 0.6 V vs two pme values shown in (c); the current densities of EOR at 0.5 V vs two pme values shown in (d); the current densities of ORR at 0.9 V vs two pme values shown in (e). The activity data in (c, e) are acquired from the ref 63 linear fits are shown with dashed black lines. (f) Schematic illustration of the degree of order at the Pt(111)-alkaline solution interface. It depicts that the electrocatalytic activity improves as the pme moves closer to the TEP of the respective reaction. Color scheme: Pt in dark gray, hydrogen in red, oxygen in blue, and small cations (Li^+^ and Na^+^) in orange and large cations (K^+^ and Cs^+^) in purple.

These consistent trends across five distinct reactions, each involving unique intermediates and covering a wide potential range, strongly highlight the critical role of interfacial entropy in governing electrocatalytic activity. The universality of this behavior suggests that the pme captures fundamental properties of the interfacial solvent environment rather than being specific to particular adsorbates. This is further supported by the presence of two distinct pme values across all cations studied, particularly evident in potential regions dominated by water reorientation, which provides direct evidence that the pme primarily reflects the entropy of the solvent network. Although the pme is measured under nonfaradaic conditions, it characterizes the underlying dynamic water structure that persists even when the electrode surface is covered by reactive intermediates under operational conditions.

The link between high interfacial entropy and enhanced kinetics can be understood within the framework of Marcus theory for electron transfer.^71^ The reorganization energy (λ) of the interfacial water layer constitutes a universal kinetic barrier for charge-transfer processes. A higher interfacial entropy, as signaled by the pme, corresponds to a more disordered and dynamically flexible water network. We propose that when the electrode potential is near the pme, a state associated with maximum solvent disorder and fluctuation, the solvent is effectively “preorganized” in a manner that lowers the reorganization energy barrier. The enhanced thermal motion and structural flexibility of water molecules facilitate the rearrangement required to stabilize the transition state, thereby accelerating reaction kinetics. This effect is most pronounced when the pme is close to the reaction’s equilibrium potential, where the driving force is small and the rate becomes highly sensitive to the reorganization barrier. The consistent cation-dependent shifts in pme and their strong correlation with activity trends across diverse reactions demonstrate that this descriptor captures a fundamental aspect of the electrified interface that governs electrocatalytic rates, largely independent of the specific adsorbates or reaction pathways involved.

Recent computational work^72^ quantifying the interfacial formation entropy for different metal–water interfaces provides strong validation of our conclusions, showing that interfacial entropy is an intrinsic thermodynamic property that correlates strongly with adsorption energetics and water structure. Our experimental approach, which measures the pme across various electrolytes, offers a direct way to probe this entropy under realistic electrochemical conditions. Together, these computational and experimental results form a synergistic picture: theory identifies the underlying principle, while our method provides an experimentally accessible descriptor. This combined perspective yields several key implications. First: decoupling electronic and solvent effects. Mapping pme for different surfaces can identify catalysts where high activity stems not just from optimal adsorption strength, but from an entropy-rich solvent environment that lowers the reorganization barrier for charge transfer, as discussed in our response regarding Marcus theory. Second: rational interface engineering. It suggests that activity can be optimized by engineering the interface to maximize beneficial entropy. For example, by tailoring the surface nanostructure, defects or hydrophilicity to promote a more disordered and dynamic solvent environment, or by selecting electrolyte cations, as shown in our work, anion or additives that restructure interfacial water. Third: integration into screening. The pme, being both experimentally accessible and computationally predictable, can complement adsorption energy descriptors in catalyst discovery, leading to the identification of materials that combine favorable electronic structure with a kinetically flexible solvent environment.

Overall, the integration of our findings with this theoretical framework positions interfacial entropy as a missing link connecting surface chemistry to solvent dynamics. By designing explicitly for this property, electrocatalyst development can evolve beyond a narrow focus on adsorbate binding toward a comprehensive strategy that also optimizes the dynamic solvent environment for superior performance. This work establishes the pme as a robust and universal descriptor of electrocatalytic activity, opening avenues for the rational design of efficient electrochemical systems through targeted interfacial engineering.

## Conclusions

In summary, we conducted a comprehensive study on the correlation between the interfacial properties and the kinetics of electrocatalytic reactions at the Pt(111)-electrolyte interface in alkaline media. The activity of multiple reactions was found to be linearly dependent on interfacial entropy, emphasizing that it can be enhanced as the pme aligns more closely with the TEP of the respective reaction. We proposed the interfacial entropy as a general descriptor for reaction activity, offering a promising approach to optimize electrocatalytic performance. Through a combination of in situ advanced techniques, we identified the critical role of alkali metal cations in the interfacial structure and characteristics by integrating their geometric, electronic, and hydration effects into a unified framework. Specifically, our analysis of *C*_dl_ demonstrated that cations with different sizes and electronegativities impact their solvation shells and interactions with adsorbates to different extents, thereby altering the cation-catalyst distance and the connectivity of the H-bond network. By quantitatively determining interfacial parameters such as the E_Cdl,min_ and pme, we found that cations also affect the interfacial electric field and water structure. Two distinct pme values exist at the interface, with each corresponding to different water molecule reorientation processes. These findings suggest that the nature of alkali metal cations is crucial in modifying the interfacial microenvironment and ultimately controlling reaction activity. Our results provide valuable mechanistic insights into the interfacial phenomena and reaction kinetics, laying the groundwork for developing strategies to optimize the electrode–electrolyte interface. This, in turn, could significantly enhance electrocatalytic performance for a broad range of reactions, driving advancements in renewable energy conversion and storage technologies.

## Supplementary Material



## Notes

Detailed experimental section; Pt single crystal surface; Additional analysis of EIS data at different potentials; Details on interfacial parameters and activity measurements (PDF)
