# Supplementary material for: Cation-Dependent Interfacial Properties Determine the Activity of Pt(111) Electrodes in Alkaline Media
Source: ACS Catal. 2025 Nov 12;15(22):19721–30. doi: 10.1021/acscatal.5c05622 (PMC12645477; doi:10.1021/acscatal.5c05622)
Supplement: Supplementary file 1 [file cs5c05622_si_001.pdf]

Supporting Information:

Cation-Dependent Interfacial Properties Determine the Activity of Pt(111) Electrodes in Alkaline Media

Haiting Yu[a], Song Xue[a,b], Elena L. Gubanova[a], Jian Zhou[a], Rodrigo Bautista[a], Adrian V. Himmelreich[a], Aliaksandr S. Bandarenka[a, c,]*

[a] Physics of Energy Conversion and Storage, Department of Physics, Technical University of Munich, James-Franck-Str. 1, 85748 Garching bei München, Germany

[b] Research Center on Advanced Chemical Engineering and Energy Materials, China University of Petroleum (East China), Qingdao 266580, P. R. China (present address)

[c] Catalysis Research Center TUM, Ernst-Otto-Fischer-Str. 1, 85748 Garching bei München, Germany

* Corresponding Author: bandarenka@ph.tum.de (A.S. Bandarenka)

Experimental Section

Cell preparation. Prior to the electrochemical measurements, all glassware and Teflon components were carefully cleaned in a Piranha solution, consisting of a 3:1 ratio mixture of 98% H_2SO_4 (Suprapur, Merck,Germany) and 30% H_2O_2 (Suprapur, Merck, Germany), followed by multiple rinsing with hot ultrapure water (18.2 MΩ·cm, Merck Millipore, Germany) to ensure complete removal of contaminants. The measurements were performed in a standard three-electrode configuration cell, which was composed of a working electrode (WE), a Pt wire (ø: 0.5 mm, 99.99% purity, MaTeck, Germany) as counter electrode (CE) and a mercury-mercurous sulfate (MMS) (SI Analytics, Germany) electrode as reference electrode (RE). The experiments were controlled using a VSP-300 potentiostat (BioLogic, France), and all reported potentials were converted to the reversible hydrogen electrode (V vs. RHE) scale for consistency and comparability.

Electrode preparation. A Pt(111) single-crystal disk electrode (ø of 10 mm, 5N, MaTecK, Jülich, Germany) was employed in this study. The electrolyte for the acidic medium was prepared using a diluted perchloric acid solution (70% $HClO_4$, Suprapur, Merck, and extra pure, Acros, Germany). Prior to each experiment, the electrode first underwent electrochemical cleaning in 0.1 M $HClO_4$ cycling between 0.12 and 1.65 V at a scan rate of 50 mV s^{-1}. Then the crystal was annealed in an isobutane flame and subsequently cooled in a mixture of 1000 ppm of CO (4.7, Air Liquide, Germany) and Ar (4.7, Air Liquide, Germany). The quality of the single-crystalline surface was verified by cyclic voltammetry (CV) in Ar-saturated 0.1 M $HClO_4$.

Electrochemical impedance measurements. EIS measurements were conducted over an AC-probing frequency range from 300 kHz to 1 Hz, with a perturbation amplitude of 10 mV. The potential step width was set to 25 mV. A shunt capacitor (~10 μF) was placed between the reference and counter electrodes to mitigate potential artifacts introduced by the potentiostat at higher frequencies. Impedance spectra were analyzed using homemade software (EIS Data Analysis 2.1) by fitting the data to an equivalent electric circuit (EEC). The quality of the fits was verified using a Kramers–Kronig check. The Nyquist plots, presented in Figure S1, recorded for the four different electrolytes at their respective pzc, confirm that the chosen EEC model provides an excellent fit to the acquired experimental spectra, with root-mean-square deviations of less than 3%.

In situ laser-induced current transient experiments. A Quanta-Ray INDI Series pulsed Nd:YAG laser (Spectra Physics Lasers, USA) was employed to generate laser pulses at a wavelength of 532 nm, featuring a 9 mm beam diameter. Each pulse lasted between 5 and 8 nanoseconds, with a repetition rate of 10 Hz. To minimize the risk of electrode surface damage, the laser intensity was attenuated using a motorized beam splitter attenuator (VA-CB-532-CONEX, Newport Corporation, USA). The resulting low-intensity beam was directed through a 30 mm diameter transparent quartz window built into a custom-fabricated three-electrode electrochemical cell, ensuring precise illumination of the electrode surface. The electrode's vertical position was adjustable to optimize beam focus. Prior to measurements, optical alignment was performed using a reduced beam power of 0.01 W. Laser-induced current transient (LICT) measurements were conducted using chronoamperometry (CA), applying potential steps of 20 mV. After holding the electrode at a fixed potential for 10 seconds to reach equilibrium, laser pulses of controlled power were manually triggered for a 4-second interval, during which the transient current responses were recorded. The more detailed descriptions can be found in ref 1.

Activity measurements.

The alkaline electrolyte solutions were prepared from LiOH (anhydrous, 99.995%, metals basis, thermo scientific, Germany), NaOH (99.99% trace metals basis, semiconductor grade, Sigma Aldrich, Germany), KOH (99.99% trace metals basis, semiconductor grade, Sigma Aldrich, Germany and CsOH (99.95% trace metals basis, monohydrate, Sigma Aldrich, Germany) and diluted to 0.1 M concentration with ultrapure water. In alkaline media, a mercury-mercurous oxide (MMO) (BAS Inc., Japan) electrode was employed as RE. The activity measurements of the electrodes were performed by recording the CV in Ar-saturated (EOR) and H_2-saturated (HER and HOR) 0.1 M alkaline solutions and measured under a hanging meniscus configuration. The measurements were performed using a Pine RDE 710 (USA) instrument, and the recorded current densities were normalized to the geometrical surface area of the electrode (0.785 cm^2).

Theory of the Laser-Induced Current Transient (LICT) Technique

Determination of the pme can be experimentally realized by the laser induced current transient method (LICT), which was reported by several groups. [2, 3, 4] The LICT technique quantifies interfacial entropy by exploiting the fundamental thermodynamic link between temperature and entropy, as expressed in the electrocapillary equation. The theoretical picture is as follows:

A short (5-8 ns), focused laser pulse irradiates the electrode surface under an applied potential, inducing a rapid and localized temperature jump (ΔT ≈ 20-40 K) at the electrode-electrolyte interface. This sudden input of thermal energy disrupts the thermodynamic equilibrium of the double layer, forcing the structured interfacial water network into a more disordered state. Subsequently, the disordered water diploes can rapidly return to the initial state and as a result of this quick relaxation, sharp current transient can be observed.

Assuming the laser pulse is uniform, the change of the temperature after the illumination can be written by:

$$\Delta T(t) = \frac{2(1-R)I}{\sqrt{\pi \kappa c d} + \sqrt{\pi \kappa_1 c_1 d_1}}\sqrt{t} \quad ((t < t_0) \qquad heating$$

$$\Delta T(t) = \frac{2(1-R)I}{\sqrt{\pi \kappa c d} + \sqrt{\pi \kappa_1 c_1 d_1}}\left[\sqrt{t} - \sqrt{t - t_0}\right] \quad ((t > t_0) \qquad Cooling$$

where κ and κ_1 are the thermal conductivity of the metal and the aqueous electrolyte, c and c_1 are the thermal capacity, d and d_1 are the density. If the time is very long, with an approximation, the temperature change reduces with increasing time:

$$\Delta T(t) = \frac{1}{2}\Delta T_0 \sqrt{\frac{t_0}{t}}$$

If the temperature change is small enough, the relationship between the potential change and temperature change can be written by:

$$\Delta E = \left(\frac{\partial E}{\partial T}\right)\Delta T = \left(\frac{\partial E}{\partial T}\right)_q \frac{1}{2}\Delta T_0 \sqrt{\frac{t_0}{t}}$$

The critical link to entropy is provided by the electrocapillary equation. [5] The measured potential shift with temperature relates directly to the entropic response:

$$\left(\frac{\partial E}{\partial T}\right)_q = -\left(\frac{\partial \Delta S}{\partial q}\right)_T$$

where ΔS is the interfacial entropy. It reaches the maximum, when $\left(\frac{\partial \Delta S}{\partial q}\right)_T$ equals to zero. This condition indicates that the change of electrochemical potential resulting from the heating of laser illumination also equals to zero. Consequently, this point identifies the potential of maximum entropy (pme) for double-layer formation. In the LICT experiment, this is observed

as the potential where the current transient vanishes, signifying that no net reorganization of the interface is required upon heating.

Since the LICT is performed potentiostatically, the system response is represented by the sharp current transients. The amplitude of the current transient is proportional to the degree of order present at the interface before the pulse. The polarity of transient indicates the net orientation of water dipoles: a negative current corresponds to a negatively charged surface where hydrogens are oriented towards the electrode, while a positive current indicates a positively charged surface where oxygens are oriented towards the electrode. The potential where this polarity undergoes a transition is the pme.

Supplementary Figures and Tables

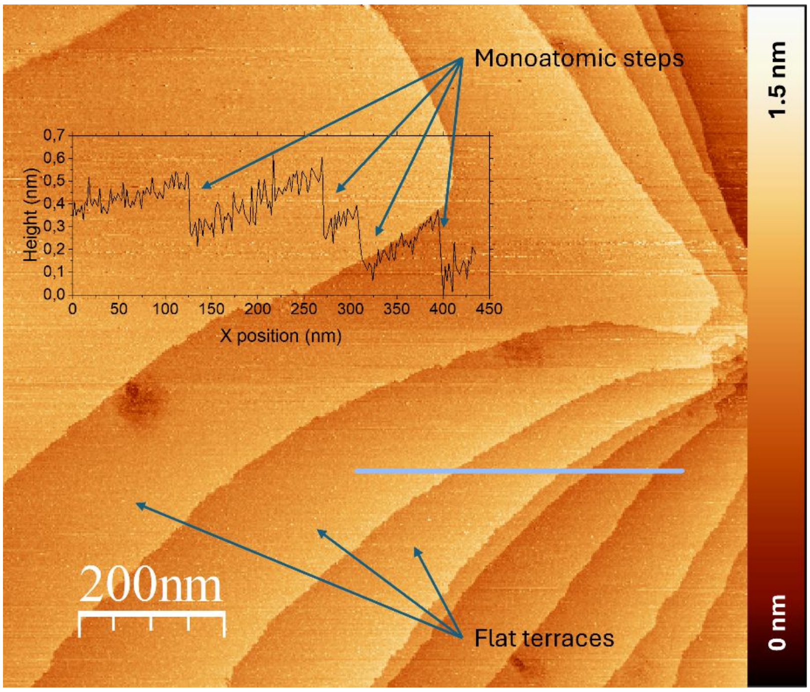

Figure S1. Electrochemical scanning tunneling microscopic image of the Pt(111) electrode surface, showing that the surface is flat enough and well prepared. The experiment was done in an Ar-saturated environment.

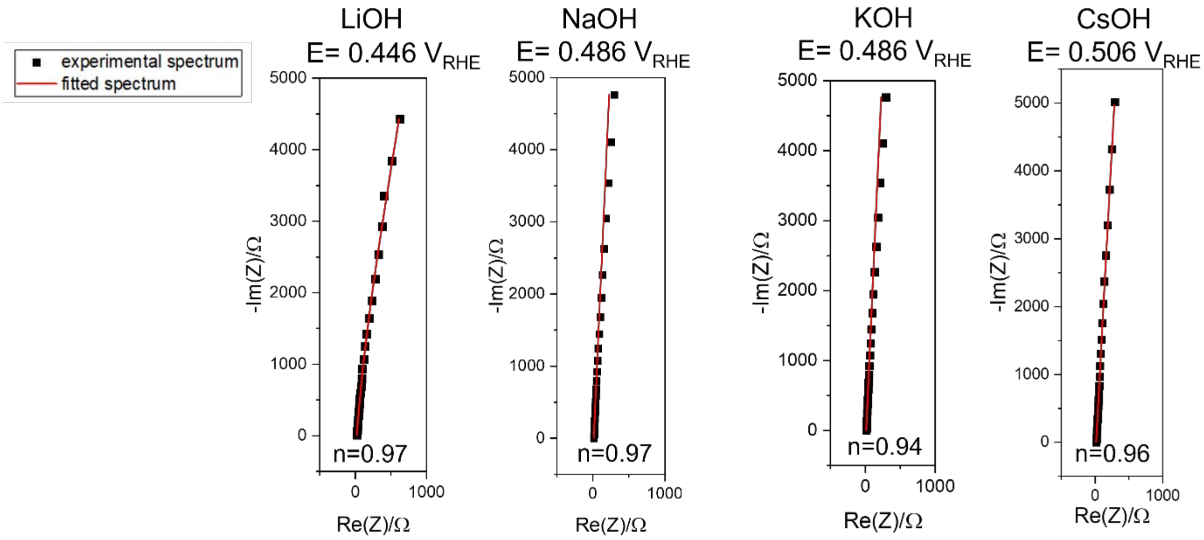

Figure S2. Examples of fitted and experimental impedance spectra in different electrolytes represented as Nyquist plots. The fitted EIS curves (solid red line) were in accordance with the experimental points (black square points). Moreover, the parameter n in the CPE was close to one ($n > 0.94$) when there were no Faradaic reactions taking place.

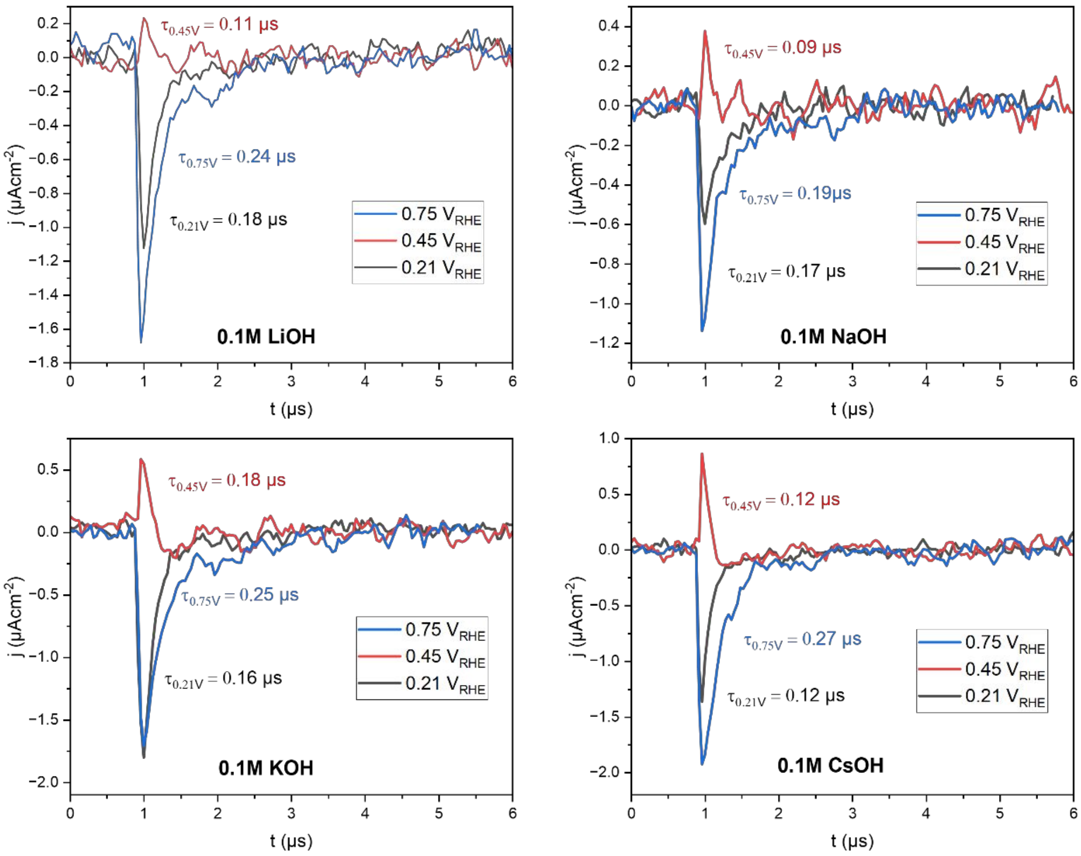

Figure S3. Representative LICT signals measured in 0.1M LiOH, NaOH, KOH, CsOH at 0.21 V_{RHE} in the H_{upd} region, 0.45 V_{RHE} in the double-layer region, and 0.75 V_{RHE} in the OH_{ad} region. The relaxation time (τ) for all transients is ~0.16 μs. This ultrafast timescale rules out slow Faradaic processes like H adsorption and confirms the signal originates from the response of the double layer, specifically water reorientation.

HER activity measurement

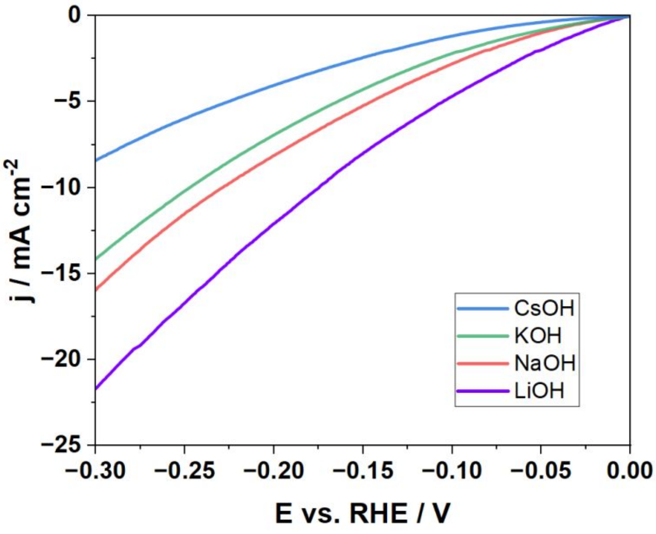

Figure S4. HER activities of Pt(111) electrode recorded in 0.1 M H_2-saturated LiOH, NaOH, KOH, and CsOH at a scan rate of 50 mV s^{-1} and a rotational speed of 1600 rpm.

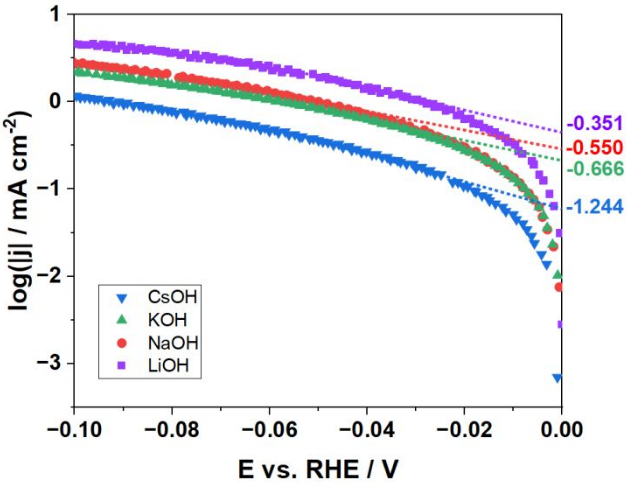

Figure S5. Logarithmic representation of current density versus potential for Pt(111) in 0.1 M alkaline solutions containing LiOH, NaOH, KOH, and CsOH. The data highlight the influence of different alkali metal cations on the electrochemical behavior, and the exchange current density j_0 can be obtained.

HOR activity measurement

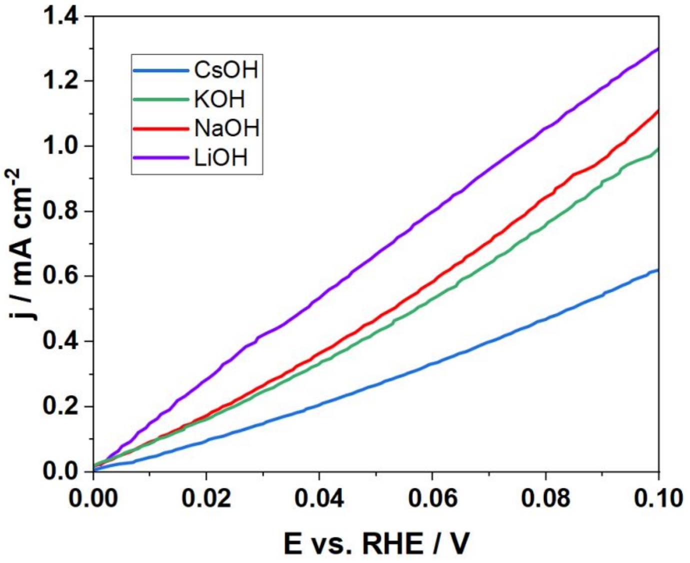

Figure S6. HOR activity measurements on Pt(111) electrode in 0.1 M H_2-saturated LiOH, NaOH, KOH, and CsOH at a scan rate of 50 mV s^{-1} and a rotational speed of 1600 rpm.

EOR activity measurement

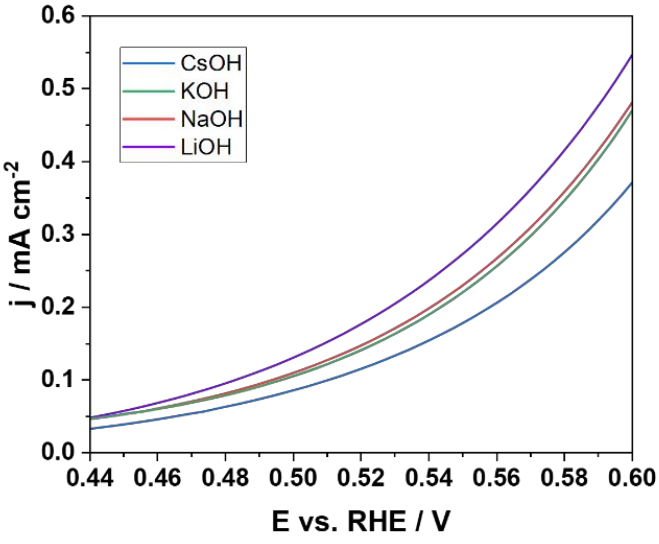

Figure S7. EOR activity measurements on Pt(111) electrode in Ar-saturated mixture solutions with 0.1 M AMOH ($AM^+ = Li^+$, Na^+, K^+, and Cs^+) + 0.1 M ethanol solution (C_2H_5OH) at a scan rate of 50 mV s^{-1}.

Table S1. Summary of Pt(111)-alkaline solution interfacial parameters including C_{dl}, $E_{Cdl,min}$ and two pmes.

Solution	$C_{dl,min}$ (μF/cm^2)	$E_{Cdl,min}$ (V vs. RHE)	pme1 (V vs. RHE)	pme2 (V vs. RHE)
LiOH	39.8±0.8	0.466	0.351 ± 0.007	0.525 ± 0.008
NaOH	48.3±2.0	0.486 ± 0.023	0.362 ± 0.006	0.567 ± 0.010
KOH	54.3±2.3	0.493 ± 0.027	0.365 ± 0.006	0.595 ± 0.001
CsOH	59.8±2.2	0.506	0.381 ± 0.012	0.623 ± 0.005

References

[1] Sarpey, T. K., Keles, E., Gubanova, E. L., & Bandarenka, A. S. Probing the electrified solid–liquid interfaces with laser-induced transient techniques. Encyclopedia of Solid-Liquid Interfaces. 2024, 43-58, 9780323856706.

[2] Benderskii, V. A., Babenko, S. D., & Krivenko, A. G. (1978). Investigation of the charge relaxation in the double layer by a thermal jump. *Journal of Electroanalytical Chemistry and Interfacial Electrochemistry*, *86*(1), 223-225.

[3] Sebastián, P., Martínez-Hincapié, R., Climent, V., & Feliu, J. M. (2017). Study of the Pt (111)| electrolyte interface in the region close to neutral pH solutions by the laser induced temperature jump technique. *Electrochimica Acta*, *228*, 667-676.

[4] Climent, V., Coles, B. A., & Compton, R. G. (2002). Laser-induced potential transients on a Au (111) single-crystal electrode. Determination of the potential of maximum entropy of double-layer formation. *The Journal of Physical Chemistry B*, *106*(20), 5258-5265.

[5] Harrison, J. A., Randles, J. E. B., & Schiffrin, D. J. (1973). The entropy of formation of the mercury-aqueous solution interface and the structure of the inner layer. *Journal of Electroanalytical Chemistry and Interfacial Electrochemistry*, *48*(3), 359-381.
